# Effect of porcine circovirus type 2 (PCV2) vaccination on PCV2-viremic piglets after experimental PCV2 challenge

**DOI:** 10.1186/1297-9716-45-13

**Published:** 2014-02-02

**Authors:** Hwi Won Seo, Changhoon Park, Kiwon Han, Chanhee Chae

**Affiliations:** 1 Department of Veterinary Pathology, College of Veterinary Medicine, Seoul National University, 1 Gwanak-ro, Gwanak-gu, Seoul 151-742, Republic of Korea

## Abstract

The objective of this study was to evaluate the effect of porcine circovirus type 2 (PCV2) vaccines on PCV2-viremic and -seropositive piglets born from naturally PCV2-infected sows against postnatal PCV2 challenge. The experimental design was aimed at mimicking commercial swine rearing conditions to evaluate the response of the PCV2 vaccine on PCV2-viremic and -seropositive piglets after experimental PCV2 challenge. PCV2a (or 2b)-viremic piglets received a PCV2 vaccine at 21 days of age followed by a PCV2b (or 2a) challenge at 49 days of age (28 days post vaccination). The PCV2 vaccines elicited a high level of humoral (as measured by immunoperoxidase monolayer assay and neutralizing antibody titers) and cellular (as measured by the frequency of PCV2-specific interferon-γ-secreting cells) immune response in the PCV2-viremic piglets after vaccination even in the presence of maternally derived antibodies (MDA). The initial infection of PCV2 in the pigs was not affected by PCV2 vaccination, however the challenging PCV2 was reduced by PCV2 vaccination on PCV2-viremic pigs. The results from this study demonstrate that the PCV2 vaccine used in this study is effective at reducing PCV2 viremia and lymphoid PCV2 DNA, even for PCV2-viremic pigs with passively acquired MDA at the time of vaccination.

## Introduction

Porcine circovirus type 2 (PCV2), a small, non-enveloped, single stranded circular DNA virus belonging to the genus *Circovirus* of the family *Circoviridae*[1], is the causative agent of several diseases and syndromes, collectively referred to as porcine circovirus-associated disease (PCVAD) [2]. Among these conditions, postweaning multisystemic wasting syndrome (PMWS) is the most important [3]. PCVAD has now been recognized worldwide and is considered to be an economically important global disease. Today, most farms use a PCV2 vaccine for the control of PCVAD since the first introduction of the PCV2 vaccine in 2006 [4].

Epidemiologic surveys have reported that 33-40% of newborn and pre-suckle piglets are PCV2-viremic [5,6]. The PCV2-viremic piglets from PCV2-infected sows were able to develop PMWS during the postnatal period if they were postnatally infected with porcine parvovirus [7]. In addition, piglets from PCV2-viremic sows were at high risk for developing PCVAD at any time throughout their life [8]. These results suggest that PCV2-viremic piglets may be immune-compromised, resulting in impaired PCV2 immunizations at postnatal periods. Consequently, immune-compromised PCV2-viremic piglets may be one of the contributing factors for increasing reports of apparent vaccine failure in late finisher pigs.

The vaccination of sows has been shown to reduce the prevalence of PCV2 viremia in their piglets under field conditions [9]. Additionally, vaccination of the sow was able to protect piglets against a PCV2 challenge up to 8 weeks of age [10]. This may explain why piglet vaccination is likely to become more popular among pig producers worldwide [4]. The results from one experimental study indicated that PCV2 vaccines can be efficacious in PCV2-viremic pigs against triple challenged with PCV2, porcine reproductive and respiratory syndrome virus (PRRSV) and porcine parvovirus (PPV) [11]. However, this study is focused on the evaluation of the efficacy of the PCV2 vaccine against triple challenge rather than the effect of PCV2 vaccination on PCV2-viremic pigs. Hence, this study aimed to evaluate the effect of PCV2 vaccines on PCV2-viremic pigs from naturally PCV2-infected sows against postnatal PCV2 challenge. To conduct this experiment, the vaccinated PCV2a (or 2b)-viremic pigs were challenged with PCV2b (or 2a) to differentiate initial PCV2 infection from challenging virus.

## Materials and methods

### Animals

A total of 105 colostrum-fed, cross-bred, conventional piglets were purchased at 7 days of age, derived from 20 sows on a commercial farm. Gilts and sows were not vaccinated for PCV2 on this farm. Clinical signs indicative of PCVAD had not been observed on the farm. Upon arrival, all pigs were tested and found to be negative for PRRSV and *Mycoplasma hyopneumoniae* according to routine serological testing.

Among the 105 pigs, 84 pigs were PCV2 seropositive and either PCV2a- or PCV2b-viremic. All PCV2-viremic pigs were the same PCV2 type as their dam. PCV2-viremic and -seropositive piglets had positive immunoperoxidase monolayer assay (IPMA) titers (ranging from 8 to 12 log_2_) for the detection of total PCV2 antibodies, neutralizing antibody (NA) titers (ranging from 7.0 to 9.0 log_2_), and genomic copies of PCV2 DNA load in the blood (ranging from 3.5 to 4.5 log_10_ PCV2 DNA copies/mL). The PCV2-viremic and -seropositive pigs used in this study had similar PCV2 viremia and serological profiles to naturally infected piglets (mean log_10_ PCV2 DNA copies ranging from 3.75 to 4.58 [5] and mean log_2_ IPMA titers ranging from 10 to 12 [12]). Twenty-one pigs were non-PCV2-viremic and seronegative for PCV2. The pigs were blocked into PCV2a, PCV2b and negative groups prior to randomization and housed separately within the facility in an environmentally controlled building as previously described [13].

### PCV2 vaccines

Commercial and experimental PCV2 vaccines were used in this study. The commercial PCV2 vaccine is an inactivated chimeric PCV1-2 vaccine (Fostera PCV, Zoetis, Madison, NJ, USA). The experimental inactivated PCV2 vaccine contained inactivated PCV2b (at a titer of 10^6^ fluorescent antibody infectious dose_50_/mL) and an aluminum hydroxide gel adjuvant (10% of volume in 1 mL/dose). PCV2 vaccines were used and administered according to the manufacturer’s instructions with regards to time and route of injection (intramuscularly in the right side of the neck).

### Experimental design

The experimental design is summarized in Table 1. A total of 105 pigs were randomly divided into 15 groups (7 pigs per group). Two groups of PCV2a-viremic pigs (groups 1 and 3) and PCV2b-viremic pigs (groups 7 and 9) were immunized with an inactivated chimeric PCV1-2 vaccine administered as a 2.0 mL dose at 21 days of age based on the manufacturer’s recommendations. Another 2 groups of PCV2a-viremic pigs (groups 2 and 4) and PCV2b-viremic pigs (groups 8 and 10) were immunized with an experimental inactivated PCV2 vaccine administered intramuscularly as a 1.0 mL dose at 21 days of age.

**Table 1:** **Study design with exposed, vaccination, and challenge statuses for PCV2 at different days post challenge (dpc)**^**a**^

**Group**	**Viremia (-42 dpc)**		**Vaccination (-28 dpc)**		**Challenge (0 dpc)**	
	**PCV2a**	**PCV2b**	**Vaccine A**^**b**^	**Vaccine B**^**c**^	**PCV2a**	**PCV2b**
1	+	-	+	-	-	+
2	+	-	-	+	-	+
3	+	-	+	-	-	-
4	+	-	-	+	-	-
5	+	-	-	-	-	+
6	+	-	-	-	-	-
7	-	+	+	-	+	-
8	-	+	-	+	+	-
9	-	+	+	-	-	-
10	-	+	-	+	-	-
11	-	+	-	-	+	-
12	-	+	-	-	-	-
13	-	-	-	-	+	-
14	-	-	-	-	-	+
15	-	-	-	-	-	-

^a^There were seven animals in each group, and necropsy was performed at 21 dpc (70 days of age) in all cases.

^b^Vaccine A: inactivated chimeric PCV1-2 vaccine (Fostera PCV, Zoetis, Madison, NJ, USA).

^c^Vaccine B: experimental inactivated PCV2 vaccine.

At 49 days of age (0 days post challenge (dpc)), the PCV2a-viremic pigs (groups 1, 2 and 5) and PCV2b-viremic pigs (groups 7, 8, and 11) were challenged intranasally with 2 mL of PCV2b (strain SNUVR000463 (GenBank no. KF871068); 5^th^ passage; 1.0 × 10^5^ tissue culture infective dose of 50% (TCID_50_)/mL) or PCV2a (strain SNUVR000032 (GenBank no. KF871067); 5^th^ passage; 1.0 × 10^5^ TCID_50_/mL), respectively. The non-PCV2-viremic pigs in groups 13 and 14 remained unvaccinated and were challenged with PCV2a or PCV2b at 49 days of age. The non-PCV2-viremic pigs in group 15 remained unvaccinated and unchallenged, and they served as the negative control group.

Blood was collected at -42, -28, 0, 7, 14, and 21 dpc. For euthanasia, pigs were sedated by an intravenous injection of sodium pentobarbital and then euthanized by electrocution [14]. Superficial inguinal lymph nodes were collected for histopathology and in situ hybridization (ISH). Methods used in this study were approved by the Seoul National University Institutional Animal Care and Use Committee.

### Quantification of PCV DNA in blood

DNA was extracted from serum samples using the QIAamp DNA mini kit. DNA extracts were used to quantify PCV2a and PCV2b genomic DNA copy numbers by real-time PCR as previously described [15].

### Serology

IPMA and NA tests were performed using both a challenging PCV2a and PCV2b as previously described [16,17].

### Enzyme-linked immunospot (ELISPOT) assay

The PCV2a and PCV2b antigens were prepared as previously described [8] and used as stimuli. The numbers of PCV2-specific interferon-γ-secreting cells (IFN-γ-SC) were determined in peripheral blood mononuclear cells (PBMC) as previously described [18,19].

### Histopathology and in situ hybridization

For the morphometric analysis of histopathological changes in lymph nodes, three superficial inguinal lymph node sections were examined “blindly” as previously described [20]. Genotype-specific in situ hybridization (ISH) was used to detect PCV2a and PCV2b, respectively, in formalin-fixed, paraffin-embedded tissues [21]. Morphometric analysis of ISH was carried out as previously described [13].

### Statistical analysis

Summary statistics were calculated for all groups to assess the overall quality of the data, including normality. The values of genomic copies of serological data and PCV2 viremia were transformed log_2_ and log_10_, respectively, prior to analysis. Continuous data (genomic copies of PCV2 DNA, serology and PCV2-specific IFN-γ-SC, number of in situ hybridization positive cells) were analyzed with a repeated measures analysis of variance (ANOVA). If the repeated measures ANOVA showed a significant effect, a one-way ANOVA with pairwise testing using the Tukey’s adjustment was performed at each time point. If the distribution of variables was not normal, the non-parametric Kruskal-Wallis and Mann-Whitney tests were used to analyze the data. Discrete data (lymphoid lesion score) were analyzed by Chi-square and/or the Fisher’s exact test. A linear regression was performed to determine the correlation between PCV2 antibody titer at the day of vaccination (21 days of age) and the increment of PCV2 antibody titer at 28 days post vaccination (delta value, defined as PCV2 antibody titer at 28 days post vaccination minus PCV2 antibody titer at the day of vaccination).

## Results

### PCV2 DNA in sera from PCV2a-viremic pigs

**Figure 1:**
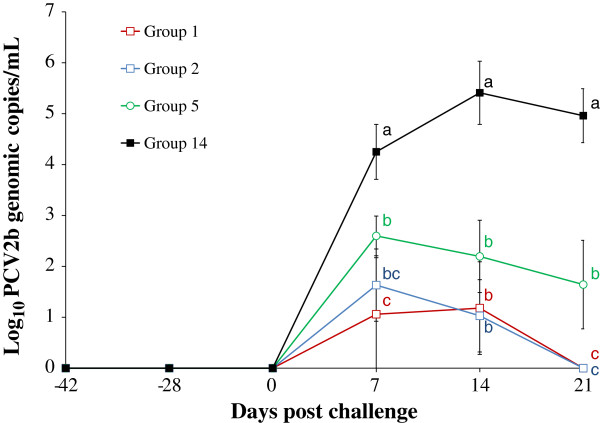
**Quantification of PCV2b DNA in sera.** Mean values of the genomic copy number of PCV2b DNA in serum from four different groups (commercial inactivated chimeric PCV1-2 vaccinated PCV2b-challenged PCV2a-viremic pigs (group 1), experimental inactivated PCV2 vaccinated PCV2b-challenged PCV2a-viremic pigs (group 2), unvaccinated PCV2b-challenged PCV2a-viremic pigs (group 5), and unvaccinated PCV2b-challenged non-PCV2-viremic pigs (group 14)) at different days post challenge. Different letters (a, b, and c) indicate statistically significant differences (*P* < 0.05) among groups.

The results of PCV2b DNA in the blood from PCV2a-viremic pigs are summarized in Figure 1. PCV2a DNA was detected in the blood of PCV2a-viremic pigs throughout the experimental study. The PCV2a-viremic pigs that were vaccinated and then challenged with PCV2b (groups 1 and 2) had a significantly (*P* < 0.05) lower number of genomic copies of PCV2b DNA in the blood compared to the unvaccinated PCV2b-challenged PCV2a-viremic pigs (group 5) and the unvaccinated PCV2b-challenged non-PCV2-viremic pigs (group 14) at 7 and 21 dpc (Figure 1). There was no significant difference in the log_10_ transformed genomic copy numbers (ranging from 3.27 to 4.12 log_10_ PCV2a DNA copies/mL) of PCV2a DNA in PCV2a-viremic pigs regardless of PCV2 vaccination and challenge among the 6 groups (groups 1, 2, 3, 4, 5, and 6). No PCV2b was detected in the blood of unvaccinated unchallenged PCV2a-viremic (group 6) and non-PCV2-viremic negative control pigs (group 15).

### PCV2 DNA in sera from PCV2b-viremic pigs

**Figure 2:**
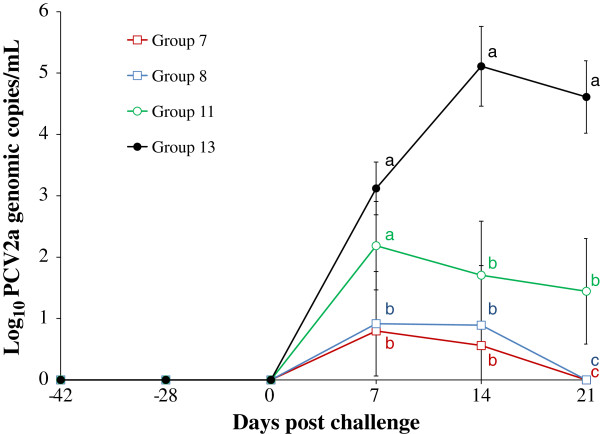
**Quantification of PCV2a DNA in sera.** Mean values of the genomic copy number of PCV2a DNA in serum from four different groups (commercial inactivated chimeric PCV1-2 vaccinated PCV2a-challenged PCV2b-viremic pigs (group 7), experimental inactivated PCV2 vaccinated PCV2a-challenged PCV2b-viremic pigs (group 8), unvaccinated PCV2a-challenged PCV2b-viremic pigs (group 11), and unvaccinated PCV2a-challenged non-PCV2-viremic pigs (group 13)) at different days post challenge. Different letters (a, b, and c) indicate statistically significant differences (*P* < 0.05) among groups.

The results of PCV2a DNA in the blood from PCV2b-viremic pigs are summarized in Figure 2. PCV2b DNA was detected in the blood of PCV2b-viremic pigs throughout the experimental study. The PCV2b-viremic pigs that were vaccinated and then challenged with PCV2a (groups 7 and 8) had a significantly (*P* < 0.05) lower number of genomic copies of PCV2a DNA in the blood than the unvaccinated PCV2a-challenged PCV2b-viremic pigs (group 11) and the unvaccinated PCV2a-challenged non-PCV2-viremic pigs (group 13) at 7, 14, and 21 dpc (Figure 2). There was no significant difference in the log_10_ transformed genomic copy numbers (ranging from 3.11 to 4.32 log_10_ PCV2b DNA copies/mL) of PCV2b DNA in PCV2b-viremic pigs regardless of PCV2 vaccination and challenge among 6 groups (groups 7, 8, 9, 10, 11, and 12). PCV2a DNA was detected after 0 dpc in the blood of pigs from PCV2a-challenged groups (7, 8, 11, and 13) regardless of PCV2 vaccination. No PCV2a was detected in the blood of unvaccinated unchallenged PCV2b-viremic (group 12) and non-PCV2-viremic negative control pigs (group 15).

### Immunoperoxidase monolayer assay titers from PCV2a (or 2b)-viremic pigs

**Figure 3:**
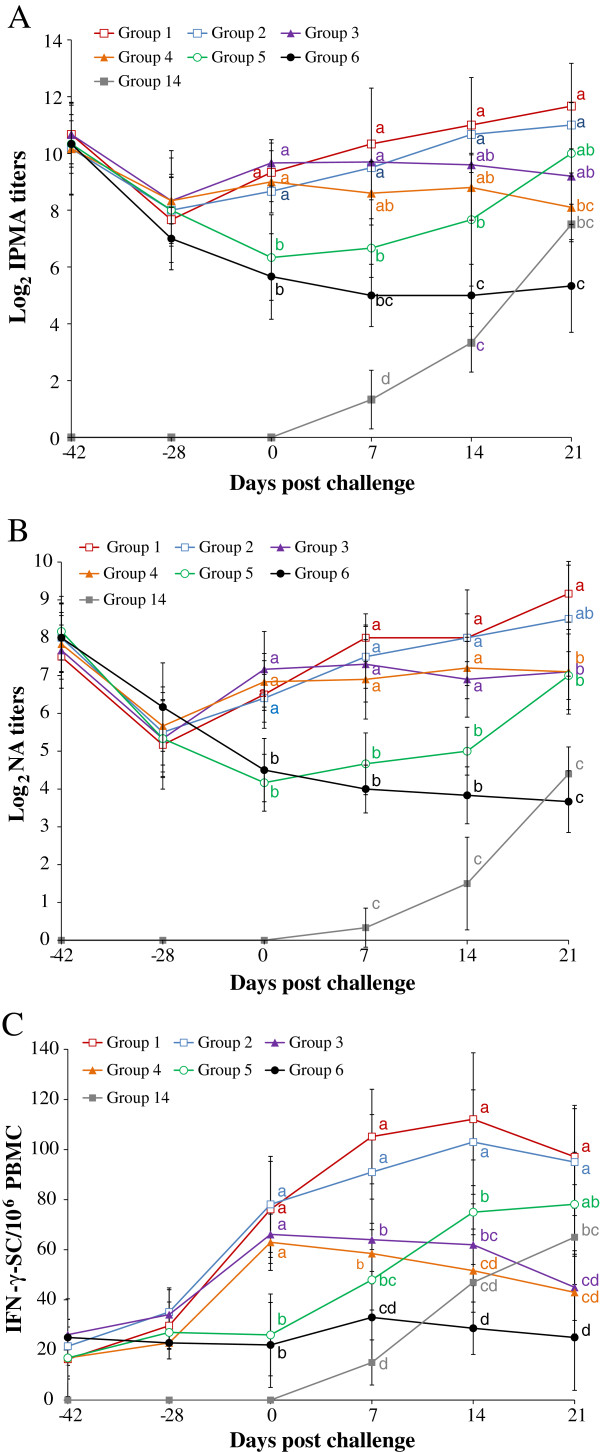
**PCV2b-specific humoral and cell-mediated immune responses.** Log_2_ transformed group means for PCV2b-specific immunoperoxidase monolayer assay (IPMA) titers **(A)**, neutralizing antibody (NA) titers **(B)**, and frequency of PCV2b-specific interferon-γ-secreting cells (IFN-γ-SC)/10^6^ peripheral blood mononuclear cells (PBMC) **(C)** from 7 different groups (commercial inactivated chimeric PCV1-2 vaccinated PCV2b-challenged PCV2a-viremic pigs (group 1), experimental inactivated PCV2 vaccinated PCV2b-challenged PCV2a-viremic pigs (group 2), commercial inactivated chimeric PCV1-2 vaccinated unchallenged PCV2a-viremic pigs (group 3), experimental inactivated PCV2 vaccinated unchallenged PCV2a-viremic pigs (group 4), unvaccinated PCV2b-challenged PCV2a-viremic pigs (group 5), unvaccinated unchallenged PCV2a-viremic pigs (group 6), and unvaccinated PCV2b-challenged non-PCV2-viremic pigs (group 14)). Different letters (a, b, c, and d) indicate statistically significant differences (*P* < 0.05) among groups.

**Figure 4:**
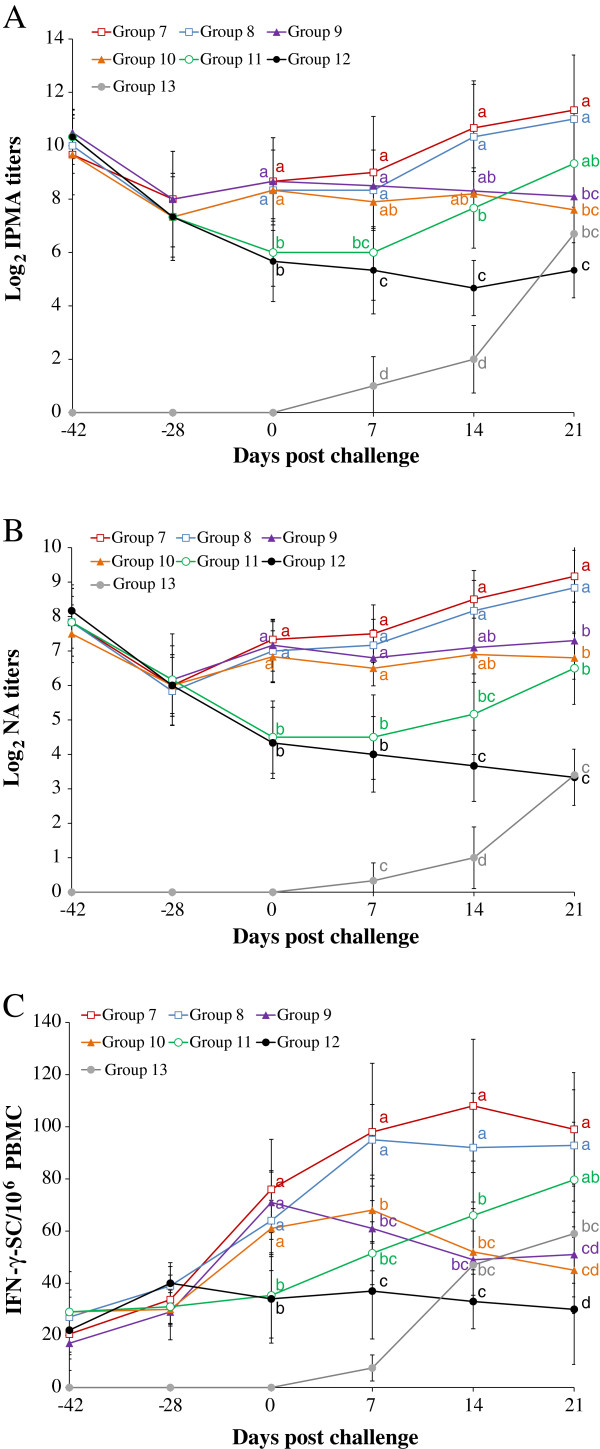
**PCV2a-specific humoral and cell-mediated immune responses.** Log_2_ transformed group means for PCV2a-specific immunoperoxidase monolayer assay (IPMA) titers **(A)**, neutralizing antibody (NA) titers **(B)**, and frequency of PCV2a-specific interferon-γ-secreting cells (IFN- γ-SC)/10^6^ peripheral blood mononuclear cells (PBMC) **(C)** from 7 different groups (commercial inactivated chimeric PCV1-2 vaccinated PCV2a-challenged PCV2b-viremic pigs (group 7), experimental inactivated PCV2 vaccinated PCV2a-challenged PCV2b-viremic pigs (group 8), commercial inactivated chimeric PCV1-2 vaccinated unchallenged PCV2b-viremic pigs (group 9), experimental inactivated PCV2 vaccinated unchallenged PCV2b-viremic pigs (group 10), unvaccinated PCV2a-challenged PCV2b-viremic pigs (group 11), unvaccinated unchallenged PCV2b-viremic pigs (group 12), and unvaccinated PCV2a-challenged non-PCV2-viremic pigs (group 13)). Different letters (a, b, c, and d) indicate statistically significant differences (*P* < 0.05) among groups.

The results of IPMA titers from PCV2a- and PCV2b viremic pigs are summarized in Figures 3A and 4A, respectively. The vaccinated PCV2b-challenged PCV2a-viremic pigs (groups 1 and 2) had significantly (*P* < 0.05) higher PCV2-specific IPMA titers than the unvaccinated PCV2b-challenged PCV2a-viremic pigs (group 5) at 0, 7, and 14 dpc, and the unvaccinated unchallenged PCV2a-viremic pigs (group 6) at 0, 7, 14, and 21 dpc (Figure 3A). The vaccinated PCV2a-challenged PCV2b-viremic pigs (groups 7 and 8) had significantly (*P* < 0.05) higher IPMA titers than the unvaccinated PCV2a-challenged PCV2b-viremic pigs (group 11) at 0, 7, and 14 dpc, and the unvaccinated unchallenged PCV2b-viremic pigs (group 12) at 0, 7, 14, and 21 dpc (Figure 4A). No PCV2-specific IPMA titers were detected in unvaccinated unchallenged non-PCV2-viremic pigs (group 15).

### Neutralizing antibody titers from PCV2a (or 2b)-viremic pigs

The results of NA titers from PCV2a- and PCV2b-viremic pigs are summarized in Figures 3B and 4B, respectively. The vaccinated PCV2b-challenged PCV2a-viremic pigs (groups 1 and 2) had significantly (*P* < 0.05) higher PCV2-specific NA titers than the unvaccinated PCV2b-challenged PCV2a-viremic pigs (group 5) at 0, 7, and 14 dpc (Figure 3B). The vaccinated PCV2a-challenged PCV2b-viremic pigs (groups 7 and 8) had significantly (*P* < 0.05) higher NA titers than the unvaccinated PCV2a-challenged PCV2b-viremic pigs (group 11) at 0, 7, 14, and 21 dpc (Figure 4B). No PCV2-specific NA titers were detected in unvaccinated unchallenged non-PCV2-viremic pigs (group 15).

### PCV2-specific interferon- γ-secreting cells from PCV2a (or 2b)-viremic pigs

The results of PCV2-specific IFN-γ-SC from PCV2a- and PCV2b-viremic pigs are summarized in Figures 3C and 4C, respectively. The vaccinated PCV2b-challenged PCV2a-viremic pigs (groups 1 and 2) had significantly (*P* < 0.05) higher mean numbers of PCV2-specific IFN-γ-SC than the unvaccinated PCV2b-challenged PCV2a-viremic pigs (group 5) at 0, 7, and 14 dpc (Figure 3C). The vaccinated PCV2a-challenged PCV2b-viremic pigs (groups 7 and 8) had significantly (*P* < 0.05) higher mean numbers of PCV2a- and PCV2b-specific IFN-γ-SC than the unvaccinated PCV2a-challenged PCV2b-viremic pigs (group 11) at 0, 7, and 14 dpc (Figure 4C). No PCV2-specific IFN-γ-SC was observed in unvaccinated unchallenged non-PCV2-viremic pigs (group 15).

### Effects of maternally derived antibodies on PCV2 vaccine seroconversion

The results show a significant negative correlation of humoral immune response with vaccination with a commercial inactivated chimeric PCV1-2 vaccine (Fostera PCV, Zoetis) in PCV2a- and PCV2b-viremic pigs (r = -0.8299, *P* = 0.017 for IPMA titers and r = -0.8467, *P* = 0.012 for NA titers) and vaccination with an experimental inactivated PCV2 vaccine in PCV2a- and PCV2b-viremic pigs (r = -0.7984, *P* = 0.023 for IPMA titers and r = -0.8208, *P* = 0.012 for NA titers).

### Lymphoid lesion and in situ hybridization score from PCV2a (or 2b)-viremic pigs

The results of lymphoid lesion and ISH score from PCV2a- and PCV2b-viremic pigs are summarized in Table 2. The pigs in six groups (group 1, 2, 3, 4, 5, and 6) had significantly (*P* < 0.05) lower lymphoid lesion scores and ISH PCV2b scores than the unvaccinated PCV2b-challenged non-PCV2-viremic pigs (group 14). The vaccinated PCV2a-challenged PCV2b-viremic pigs (groups 7 and 8) had significantly (*P* < 0.05) lower ISH PCV2a scores than the unvaccinated PCV2a-challenged PCV2b-viremic pigs (group 11). No hybridization signals for PCV2a and PCV2b were detected in lymph nodes from unvaccinated unchallenged non-PCV2-viremic pigs (group 15).

**Table 2:** Results of lymphoid lesion score and the number of in situ hybridization (ISH) positive cells for PCV2a and PCV2b at 21 dpc

**Challenge status**	**Group**	**Lymphoid lesion score**	**In situ hybridization score**	
			**PCV2a**	**PCV2b**
2a	7	0.47 ± 0.35^a,b^	0.71 ± 1.25^b^	10.42 ± 5.22^b^
	8	0.51 ± 0.42^a,b^	0.85 ± 1.46^b^	11.14 ± 5.89^b^
	11	0.71 ± 0.69^a,b^	8.14 ± 6.06^a^	10.7 ± 5.93^b^
	13	1.57 ± 0.53^c^	30.71 ± 10.96^c^	0.0 ± 0.0^a^
2b	1	0.53 ± 0.52^a,b^	9.14 ± 6.56^a^	2.0 ± 2.51^a^
	2	0.43 ± 0.53^a,b^	7.28 ± 6.87^a^	2.42 ± 3.1^a^
	5	0.86 ± 0.48^b^	8.42 ± 5.15^a^	7.14 ± 4.14^b^
	14	2.00 ± 0.81^c^	0.0 ± 0.0^b^	37.29 ± 8.96^c^
Non-challenged	3	0.29 ± 0.49^a^	5.71 ± 6.23^a^	0.0 ± 0.0^a^
	4	0.23 ± 0.41^a^	6.14 ± 5.49^a^	0.0 ± 0.0^a^
	6	0.31 ± 0.37^a^	8.57 ± 6.07^a^	0.0 ± 0.0^a^
	9	0.35 ± 0.39^a,b^	0.0 ± 0.0^b^	8.85 ± 5.17^b^
	10	0.41 ± 0.38^a,b^	0.0 ± 0.0^b^	7.14 ± 4.77^b^
	12	0.43 ± 0.49^a,b^	0.0 ± 0.0^b^	9.14 ± 5.27^b^
	15	0.0 ± 0.0^a^	0.0 ± 0.0^b^	0.0 ± 0.0^a^

Different letters (a, b, and c) indicate significant (*P* < 0.05) difference among groups.

## Discussion

The use of vaccination to immunize and protect pigs against PCV2 infection has been widely evaluated under experimental and field conditions [10,18,22-26]. Numerous experiments have shown that PCV2 vaccines are highly efficacious at protecting pigs against PCV2 infection. Ideally, PCV2 vaccination should be administered while residual maternally derived antibodies (MDA) are minimal and before pigs become naturally infected. In the field, vaccination is likely administered routinely in the face of maternal immunity and infection in the field. Field data indicate that PCV2 vaccines are not inhibited by MDA when efficacy is assessed in terms of the reduction of PCV2-associated lesions and viral load in the serum [23,25]. Hence, MDA interference does not significantly hamper vaccine efficacy [18,23,25]. However, little scientific information is available describing the effect of PCV2 vaccination on PCV2-viremic and -seropositive pigs. The PCV2-viremic and -seropositive pigs used in this study had similar PCV2 viremia and serological profiles to naturally infected piglets [5,12]. Therefore, the experimental design attempted to mimic commercial swine rearing conditions to evaluate the response of PCV2-viremic and -seropositive piglets after PCV2 vaccination and experimental PCV2 challenge.

In the present study, the vaccinated PCV2a-viremic pigs were challenged with PCV2b to differentiate initial PCV2 infection from challenging virus. PCV2 vaccines significantly reduced the amount of challenging PCV2a (or 2b) in the blood of PCV2b (or 2a)-viremic pigs that received a PCV2 vaccine and were subsequently challenged. The reduction of PCV2 viremia by the vaccine plays a critical role in controlling PCV2 infection [4]. A high PCV2 viremia has already been shown to be associated with the development of PCVAD [27-29]. Therefore, increased PCV2 viremia equates to an increased risk of developing PCVAD. The results of the present study demonstrate that PCV2 vaccination can protect PCV2-viremic pigs from a subsequent PCV2 challenge.

Despite the significant reduction of the challenging PCV2 in the blood as a result of PCV2 vaccination, PCV2a (or 2b)-viremic pigs that received a PCV2 vaccine followed by a PCV2b (or 2a) challenge retained a PCV2a (or 2b)-viremia as a result of previous exposure until the termination of the experimental study at 21 dpc. The failure to reduce or eliminate previously encountered PCV2 in the blood may result in a prolonged PCV2 viremia. It has been reported that PCV2 can maintain viremia up to 140 dpc for PCV2a and 69 dpc for PCV2b [30,31]. The prolonged previously encountered PCV2 viremia may be transmitted to susceptible animals following direct contact with pigs and can perpetuate the infection within a herd. Further study is needed to determine the effect of previous exposure to PCV2 after PCV2 vaccination.

The efficacy of PCV2 vaccination in PCV2-viremic piglets can be affected by the presence of MDA. Therefore, the PCV2-viremic piglets may face potential interference from PCV2 antibodies present at the time of vaccination. Whether the efficacy of vaccination in piglets can be affected by the presence of MDA is controversial. Piglets with high IPMA titers (> 10 log_2_) have been shown to experience interference with the development of a humoral immune response following vaccination, while piglets with moderate titers (< 8 log_2_) do not [22]. In the present study, PCV2-viremic piglets had moderate IPMA (< 8 log_2_) and NA (< 7 log_2_) titers at the time of vaccination. Moreover, the PCV2 vaccine elicited a high level of IPMA and NA titers, and the frequency of IFN-γ-SC in PCV2-viremic and -seropositive piglets was high even in the presence of MDA. These two immunological parameters are responsible for the reduction of PCV2 viremia and control PCV2 infection [28,32]. These data suggest that preexisting, moderate levels of MDA do not interfere with the development of an active immune response after PCV2 vaccination in PCV2-viremic piglets.

There were no significant differences in the frequency of PCV2-specific IFN-γ-SC in response to the whole virus when either PCV2a or PCV2b was used as a recall antigen, as demonstrated in a previous study [33]. The PCV2a-based commercial vaccine and the PCV2b-based experimental vaccine induced similar profiles of PCV2a- and PCV2b-specific IFN-γ-SC in the PCV2a- and PCV2b-viremic pigs when either PCV2a or PCV2b was used as a recall antigen. These results suggest the existence of conserved immunodominant T-cell epitopes between both genotypes, which is further supported by the cross-protection of PCV2a-based vaccines against a PCV2b challenge [34,35]. These results are clinically significant because all the commercial PCV2 vaccines currently used worldwide are based on the PCV2a genotype.

Rather than PCV2 vaccination, the reduction of PCV2b (or 2a) viremia in pigs previously exposed to PCV2a (or 2b) may be due to cross protection between the two genotypes. PCV2a- (or 2b-) viremic pigs that were challenged with PCV2b (or 2a) had significantly reduced levels of challenging PCV2 in the blood. These results were in agreement with a previous study [36]. Although cross protection between PCV2 and PCV2b exists [36], PCV2a-viremic pigs that received a PCV2 vaccine followed by a PCV2b challenge had a significantly lower number of genomic copies of challenging PCV2b in the serum than the unvaccinated PCV2a-viremic pigs that were only challenged with PCV2b. These results indicate that active immune responses by PCV2 vaccination, rather than natural infection or passively transferred MDA, play an important role in reducing PCV2b viremia in PCV2a-viremic pigs that received a PCV2 vaccine followed by a PCV2b challenge and vice versa.

PCV2-associated microscopic lesions were not prominent in this study because the pigs were challenged with PCV2 alone rather than co-challenged with other viruses, such as PRRSV or PPV. Vaccination effectively reduced the number of PCV2-associated microscopic lesions and the level of PCV2 DNA in the lymphoid tissues of PCV2-viremic piglets that received a PCV2 vaccine followed by a PCV2 challenge. Many pig producers may believe that PCV2-viremic or -seropositive piglets contribute to vaccine failure. The results from this study demonstrate that the PCV2 vaccine was effective in reducing PCV2 viremia and lymphoid PCV2 DNA even when used on PCV2-viremic pigs with passively acquired MDA at the time of vaccination.

## Competing interests

The authors declare that they have no competing interests.

## Authors’ contributions

HWS performance of the experimental trials, data analysis and writing of the manuscript, CP and KH preparation of the inoculum and lab analysis and inoculation of the virus, CC development of the protocol, design of the study, review of the final manuscript, approval for publication. All authors read and approved the final manuscript.
